# AnemoCheck-LRS: an optimized, color-based point-of-care test to identify severe anemia in limited-resource settings

**DOI:** 10.1186/s12916-020-01793-6

**Published:** 2020-11-16

**Authors:** Marina S. Perez-Plazola, Erika A. Tyburski, Luke R. Smart, Thad A. Howard, Amanda Pfeiffer, Russell E. Ware, Wilbur A. Lam, Patrick T. McGann

**Affiliations:** 1 Washington University School of Medicine, St. Louis, MO USA; 2 The Wallace H. Coulter Department of Biomedical Engineering at Georgia Tech and Emory University, Atlanta, GA USA; 3 Aflac Cancer and Blood Disorders Center of Children’s Healthcare of Atlanta and Department of Pediatrics, Emory University School of Medicine, Atlanta, GA USA; 4 The Parker H. Petit Institute for Bioengineering and Biosciences, Georgia Institute of Technology, Atlanta, GA USA; 5 Sanguina, LLC, Atlanta, GA USA; 6 Division of Hematology, Cincinnati Children’s Hospital Medical Center, 3333 Burnet Ave, MLC 11027, Cincinnati, OH 45229 USA; 7 Department of Pediatrics, University of Cincinnati College of Medicine, Cincinnati, OH USA

**Keywords:** Point-of-care diagnostics, Global health, Anemia, Hematology

## Abstract

**Background:**

Severe anemia is common and frequently fatal for hospitalized patients in limited-resource settings. Lack of access to low-cost, accurate, and rapid diagnosis of anemia impedes the delivery of life-saving care and appropriate use of the limited blood supply. The WHO Haemoglobin Colour Scale (HCS) is a simple low-cost test but frequently inaccurate. AnemoCheck-LRS (limited-resource settings) is a rapid, inexpensive, color-based point-of-care (POC) test optimized to diagnose severe anemia.

**Methods:**

Deidentified whole blood samples were diluted with plasma to create variable hemoglobin (Hb) concentrations, with most in the severe (≤ 7 g/dL) or profound (≤ 5 g/dL) anemia range. Each sample was tested with AnemoCheck-LRS and WHO HCS independently by three readers and compared to Hb measured by an electronic POC test (HemoCue 201^+^) and commercial hematology analyzer.

**Results:**

For 570 evaluations within the limits of detection of AnemoCheck-LRS (Hb ≤ 8 g/dL), the average difference between AnemoCheck-LRS and measured Hb was 0.5 ± 0.4 g/dL. In contrast, the WHO HCS overestimated Hb with an absolute difference of 4.9 ± 1.3 g/dL for samples within its detection range (Hb 4–14 g/dL, *n* = 405). AnemoCheck-LRS was much more sensitive (92%) for the diagnosis of profound anemia than WHO HCS (22%).

**Conclusions:**

AnemoCheck-LRS is a rapid, inexpensive, and accurate POC test for anemia. AnemoCheck-LRS is more accurate than WHO HCS for detection of low Hb levels, severe anemia that may require blood transfusion. AnemoCheck-LRS should be tested prospectively in limited-resource settings where severe anemia is common, to determine its utility as a screening tool to identify patients who may require transfusion.

## Background

Severe anemia, defined as hemoglobin (Hb) < 7 g/dL for children 6–59 months per WHO definitions [1], is common and associated with high mortality rates in limited-resource settings, affecting up to 30% of hospitalized patients in sub-Saharan Africa [2, 3]. The majority of cases of severe anemia are found in pregnant women and children [4, 5]. In an effort to judiciously utilize the limited global blood supply, the World Health Organization (WHO) guidelines currently recommend blood transfusion for children with Hb < 4 g/dL in all cases and for patients with Hb < 6 g/dL if there are other clinical complications [6]. For pregnant women, transfusion is recommended by the WHO when Hb < 5 g/dL [7], and this is a commonly used threshold for transfusion in limited-resource settings. Lack of timely transfusions for children with severe anemia has been associated with a significant increase in mortality in several studies within Africa [8, 9]. The accurate diagnosis of severe anemia is critical to appropriately utilize the very limited blood supply and to triage and treat patients in a timely manner to provide life-saving care.

The WHO Integrated Management of Childhood Illness manual recommends the clinical assessment of anemia in children primarily by determining the presence and severity of palmar pallor [10, 11]. If “some palmar pallor” is present, iron therapy is recommended and follow-up in 14 days. If “severe palmar pallor” is present, referral to the hospital is recommended [10]. However, the clinical assessment of anemia has been demonstrated to have highly variable sensitivity and specificity [12], and a delay in the diagnosis of severe anemia can be life-threatening. Unfortunately, the accurate and timely laboratory diagnosis of anemia is often difficult in most limited-resource settings due to lack of equipment, inadequate replenishment of reagents, poor maintenance or calibration of existing equipment, inadequately trained laboratory personnel, or unreliable and inconsistent access to electricity. Due to inaccessible or inaccurate diagnostics, the valuable and limited blood supply is often transfused inappropriately. Two studies examining transfusion practices in Uganda and Tanzania demonstrated that many blood transfusions (as many as half) were administered inappropriately, either without a Hb measurement or with Hb values for which transfusion would not be recommended [13, 14].

**Fig. 1:**
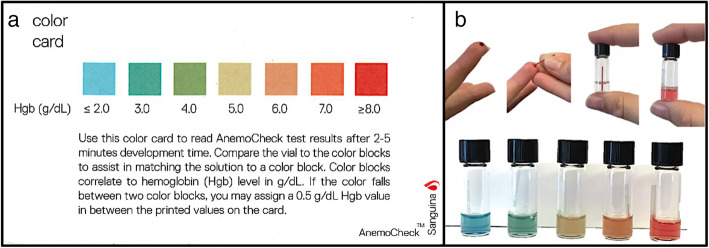
**AnemoCheck-LRS.** The AnemoCheck-LRS is a rapid, color-based test that estimates hemoglobin concentration. The addition of a drop of blood (10 μL) to a small amount of reagent changes the color of the solution. The initially clear solution changes color ranging from blue to red. The limits of detection have been specifically designed to detect severe and profound anemia with a detection range of 2–8 g/dL. Panel **a** depicts the colors that correspond with each 1 g/dL increase in hemoglobin concentration, and panel **b** detects the test performed using a drop of blood at the point of care. Two authors of this manuscript (WAL and EAT) as employees of Sanguina, Inc., are owners of the images and grant permission to re-use the images here. Image **b** is a part of the package from the manufacturer and is not from a patient

The AnemoCheck™ is a rapid, inexpensive, color-based point-of-care (POC) test initially designed to diagnose anemia in high-resource settings [15], and subsequently modified for use in limited-resource settings (called AnemoCheck-LRS) [16, 17]. A drop of blood is added to prefilled reagents to create a color change that corresponds to a specific hemoglobin level. Our previous evaluation of a prototype AnemoCheck-LRS in a field study in Tanzania demonstrated encouraging results, but we also found several opportunities for improvement and optimization of the assay [17]. Based on these results and reconsideration of the potential clinical utility of this test in the real world (outside of a research study) as a screening test for severe and profound anemia (Hb ≤ 7 g/dL and Hb ≤ 5 g/dL, respectively), the AnemoCheck assay was further optimized to more accurately detect these critical hemoglobin thresholds. The newly modified AnemoCheck-LRS test allows identification of Hb ranging from 2 to 8 g/dL, with clear color changes that are especially apparent at the 5 g/dL threshold (Fig. 1a), enabling accurate diagnosis of clinically meaningful results in settings with a high prevalence of profound anemia. The current study aimed to evaluate this optimized assay in a controlled laboratory environment to validate the accuracy of the changes prior to implementation in the field. We also aimed to compare the accuracy of the AnemoCheck-LRS to the WHO Haemoglobin Colour Scale as a POC screening tool for severe and profound anemia.

## Methods

### Study design and study objectives

This was a prospective study, with measurement of Hb concentration using three different rapid POC methods: AnemoCheck-LRS (Sanguina, LLC, Atlanta, GA), WHO Haemoglobin Colour Scale (HCS; Copack GmbH, Oststeinbek, Germany), and HemoCue Hb 201^+^ (HemoCue AB, Ängelholm, Sweden), compared to a gold standard method (ADVIA 2120i Hematology System, Siemens Healthcare Diagnostics Erlangen, Germany). The study was performed using leftover, deidentified venous blood samples collected for a variety of clinical indications and repurposed as part of an Institutional Review Board-approved research protocol. The results of the two visual POC tests (AnemoCheck-LRS and WHO HCS) were interpreted by three different readers to evaluate the reproducibility and inter-rater agreement of each test. Our primary study objective was to determine the reliability of the second-generation, optimized AnemoCheck-LRS to detect severe (Hb ≤ 7 g/dL) and profound (Hb ≤ 5 g/dL) anemia compared to other commercially available POC Hb assays.

### Point-of-care assays

The first-generation Hb POC assay AnemoCheck-LRS has previously been described and tested in both laboratory and field settings [15–17]. The test requires only a single drop of blood added to a tube of prefilled reagents. Upon mixing, the initially clear solution changes color within 1–2 min with distinct colors corresponding to a specific hemoglobin concentration. The assay relies on the reaction between hydrogen peroxide, 3,3′,5,5′-tetramethylbenzidine, and hemoglobin. The color corresponds to the Hb concentration (Fig. 1a) and enables rapid identification of anemia ranging from a Hb of ≤ 2 g/dL (light blue) to ≥ 8 g/dL (red). Following field testing in Tanzania with the prototype AnemoCheck-LRS [17], the assay was further optimized to create a sharp color distinction around the critical hemoglobin level of 5 g/dL, where transfusion may be considered.

**Fig. 2:**
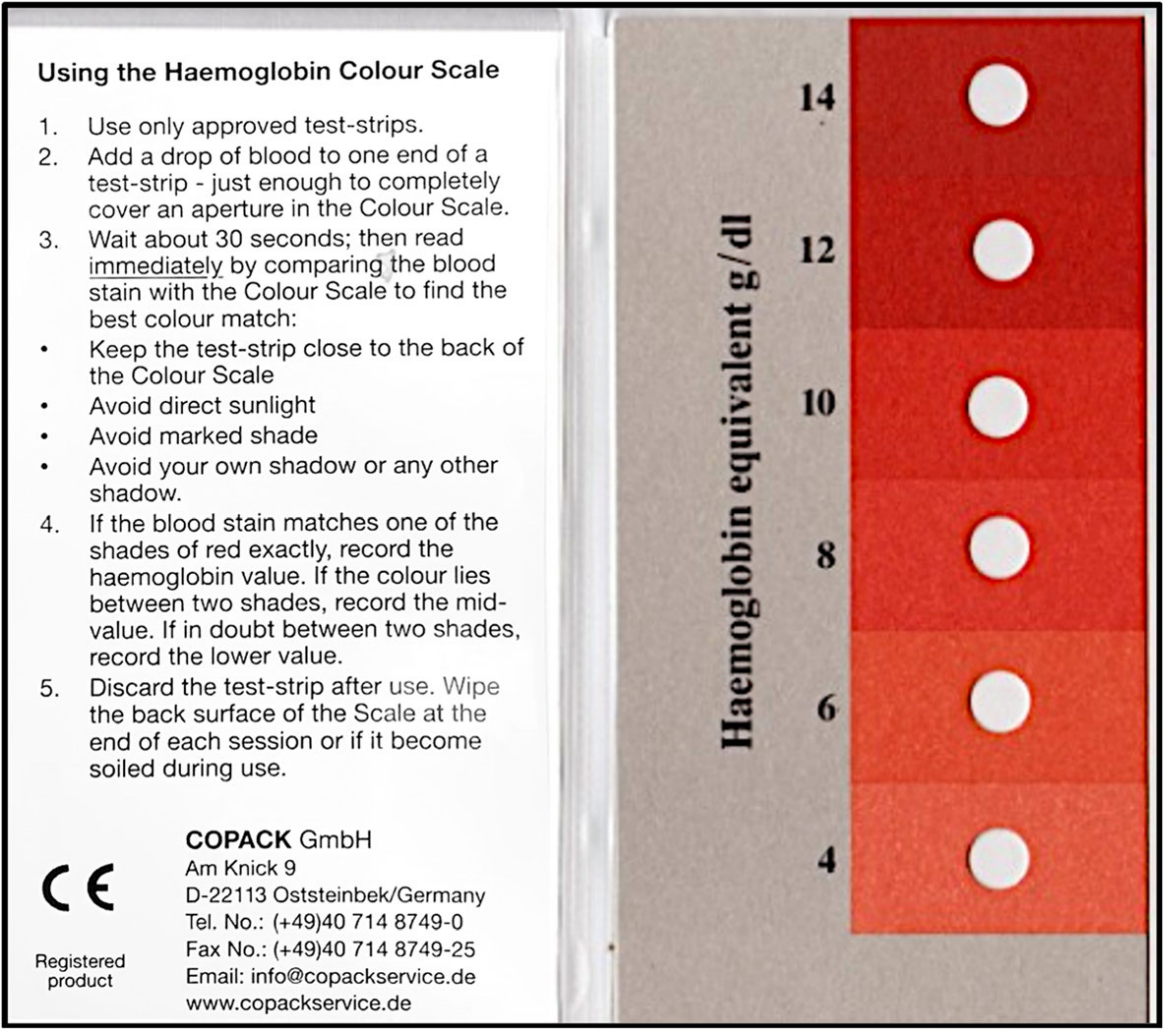
**WHO Haemoglobin Colour Scale.** The WHO Haemoglobin Colour Scale is a rapid, point-of-care diagnostic assay that estimates hemoglobin concentration ranging from 4 to 14 g/dL. A drop of blood is added to specialized filter paper, and the resulting color is compared to the depicted shades of red/pink in the figure. The photo for this figure was taken by the authors

The WHO HCS is a simple, rapid, POC test requiring a single drop of blood on special filter paper, distributed with a foldable, plastic-covered booklet containing detailed instructions for use on one page, and a red color scale with corresponding hemoglobin concentrations (4.0–14.0 g/dL) in 2 g/dL increments (Fig. 2).

The HemoCue Hb 201^+^ is a handheld, battery-operated device. A drop of blood is collected with a heparinized microcuvette containing dried reagents that enable a modified azide methemoglobin reaction to occur. The microcuvette is inserted into the handheld device that measures the hemoglobin concentration using a photometric method and displays the result on its screen.

### Point-of-care readers

In order to test the interpretability of the assays among a diverse set of end-users who have varying degrees of familiarity with the assays, 20 readers from diverse backgrounds were recruited to participate in the study. Among our readers were college students, physicians, research staff, administrative personnel, and laboratory technicians. Most had no experience using a POC test to detect anemia.

### Sample preparation

In order to prepare blood samples representative of limited-resource settings with a high prevalence of severe (Hb ≤ 7 g/dL) and profound (Hb ≤ 5 g/dL) anemia, we diluted venous blood samples to create Hb concentrations within the desired range of 2–10 g/dL. In order to robustly evaluate the accuracy of these assays to diagnose profound anemia possibly needing transfusion, the objective was to dilute samples such that 50% (100 samples) had Hb concentration ≤ 5 g/dL and 50% had Hb of 5–10 g/dL. Hemoglobin concentration of the venous blood sample was first measured using an ADVIA 2120i Hematology System. Each sample was then diluted using purchased commercial plasma or pooled plasma from leftover samples to create a 1000-μL sample with the desired hemoglobin concentration. Hemoglobin concentration of the diluted sample was then measured using the ADVIA 2120i Hematology System to confirm the success of the calculations and procedures. All pre-diluted blood samples were obtained venously, refrigerated, and used within 3 days.

### Testing procedures

All testing was conducted in a laboratory setting under both natural (by a window) and artificial (indoor, not near a window) lighting conditions. Each batch of diluted samples was created the morning of the day of testing as described above, pipetted into plastic, 1.5-mL Eppendorf tubes, and kept on a rocker at room temperature to ensure sample homogeneity. Three readers participated in each testing session and scored at least 10 samples using both the WHO HCS and the AnemoCheck-LRS assay. Each participant was read the instructions provided by each test, and a simple visual demonstration was performed to ensure consistency prior to each session. In order to avoid bias, all participants were blinded to all measured hemoglobin concentrations by HemoCue and ADVIA, and to the numbers their participant counterparts assigned to each sample.

For the AnemoCheck-LRS, exactly 10 μL of blood from the sample was added to a glass vial with 0.5 mL of prefilled AnemoCheck-LRS reagents (Fig. 1b). Next, the vial was tightly closed, gently inverted for 10–20 s to mix the contents, and placed on the laboratory bench for an additional 2 min before assessment. Each reader was allowed 30 s to record a Hb value for each sample, according to the AnemoCheck-LRS scale. This procedure was repeated for a total of 10 samples per session.

Immediately following AnemoCheck-LRS testing, 10 blood samples corresponding to the same Hb values but with different IDs were assessed using the WHO HCS. Approximately 10 μL of blood (or the equivalent of one drop of blood) was pipetted onto a WHO HCS paper testing strip for each sample. Similar to above for AnemoCheck, participants were instructed to record the perceived hemoglobin concentration according to the WHO HCS booklet (Fig. 2). Participants were instructed to take no longer than 30 s to assign a hemoglobin concentration to each sample.

After the session’s samples were assessed by participants using both AnemoCheck-LRS and WHO HCS testing, hemoglobin was also measured using the HemoCue Hb 201^+^ to determine its accuracy as a portable test that may be used in future field studies for the detection of low concentrations of hemoglobin.

### Patient and public involvement

At this stage, our work focused on the laboratory-based evaluation of the accuracy of this novel diagnostic test and did not include patients or the public. Future studies will include patient and public involvement to allow for appropriate implementation and utilization in global health settings.

### Statistical analysis

Statistical analyses were performed using Stata 13.1 (StataCorp, College Station, TX) and NCSS 2019 (NCSS, LLC, Kaysville, Utah). Pearson correlations and general linear regression models and prediction limits were used to assess associations between hemoglobin level via each POC test and the value obtained by the ADVIA 2120i. Intraclass correlation coefficients were calculated to assess agreement between each of the 3 readers for each POC test. Bland-Altman plots were created to assess differences between each POC test and the gold standard at different hemoglobin levels. Receiver-operating curves were also created to assess whether AnemoCheck-LRS threshold could be used to improve the sensitivity of the test as a screening tool to identify profound anemia (Hb ≤ 5 g/dL). Sensitivities, specificity, and both positive and negative predictive values were obtained from the ROC analyses.

## Results

### Samples

A total of 200 individual samples were prepared as described above with close approximation to target hemoglobin. The mean ± SD final hemoglobin concentration of the diluted samples was 5.2 ± 1.9 g/dL, ranging from 1.7 to 10.1 g/dL. The hemoglobin concentration for most samples (80%) was ≤ 7.0 g/dL, which meets the upper threshold of severe anemia as defined by the WHO, and half (50%) had Hb ≤ 5 g/dL, defined in this study as profound anemia. Each sample had three independent scores for each of the POC tests (WHO HCS and AnemoCheck-LRS) for a total of 600 results for each visual POC test. The median number of samples scored per reader was 20 (range 10–200).

### Accuracy of AnemoCheck-LRS

**Fig. 3:**
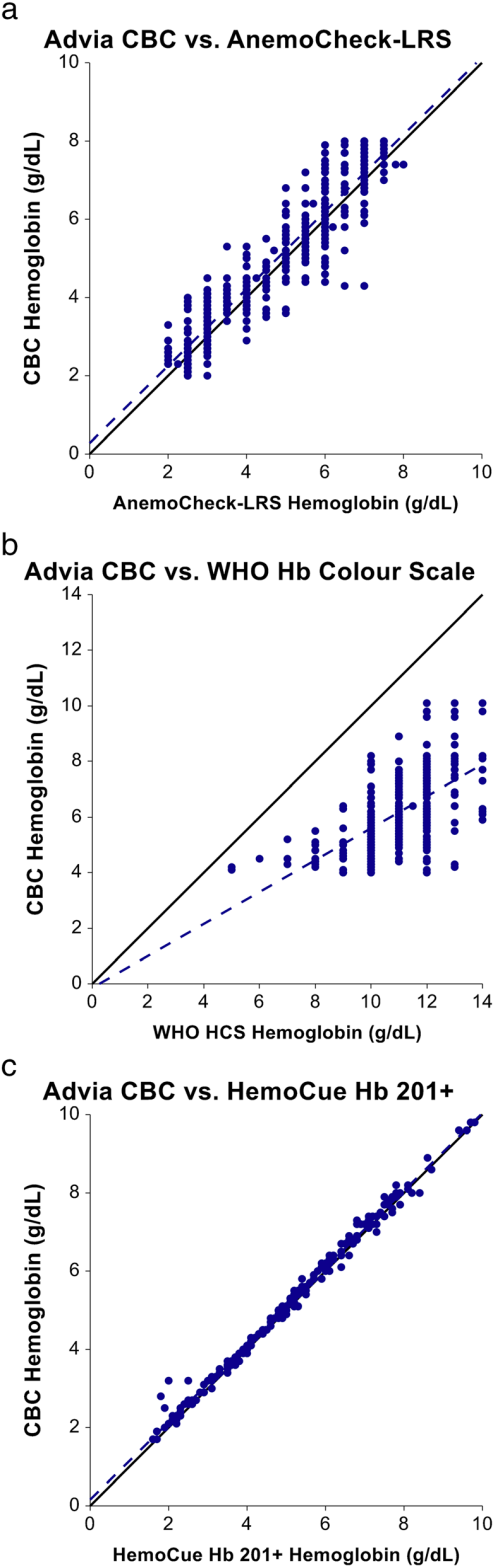
**Accuracy of rapid hemoglobin tests.** Panel **a** illustrates a very strong positive correlation between the AnemoCheck-LRS and the gold standard method (*r* = 0.93, *p* < 0.0001) for samples within the detection limit of the assay (Hb 2–8 g/dL); panel **b** illustrates that the WHO Haemoglobin Colour Scale (WHO HCS) consistently overestimated hemoglobin concentration compared to the gold standard method with a moderate correlation (*r* = 0.57, *p* < 0.0001) for samples within the detection limit of the assay (Hb 4–14 g/dL); panel **c** illustrates the very strong correlation of the HemoCue Hb 201^+^ test to the gold standard method (*r* = 0.99, *p* < 0.0001) which was included as a POC method for the study to evaluate its future utility in the field as a portable, replacement gold standard. For each panel, the solid line represents a perfect agreement and the dotted line represents a best fit line

**Fig. 4:**
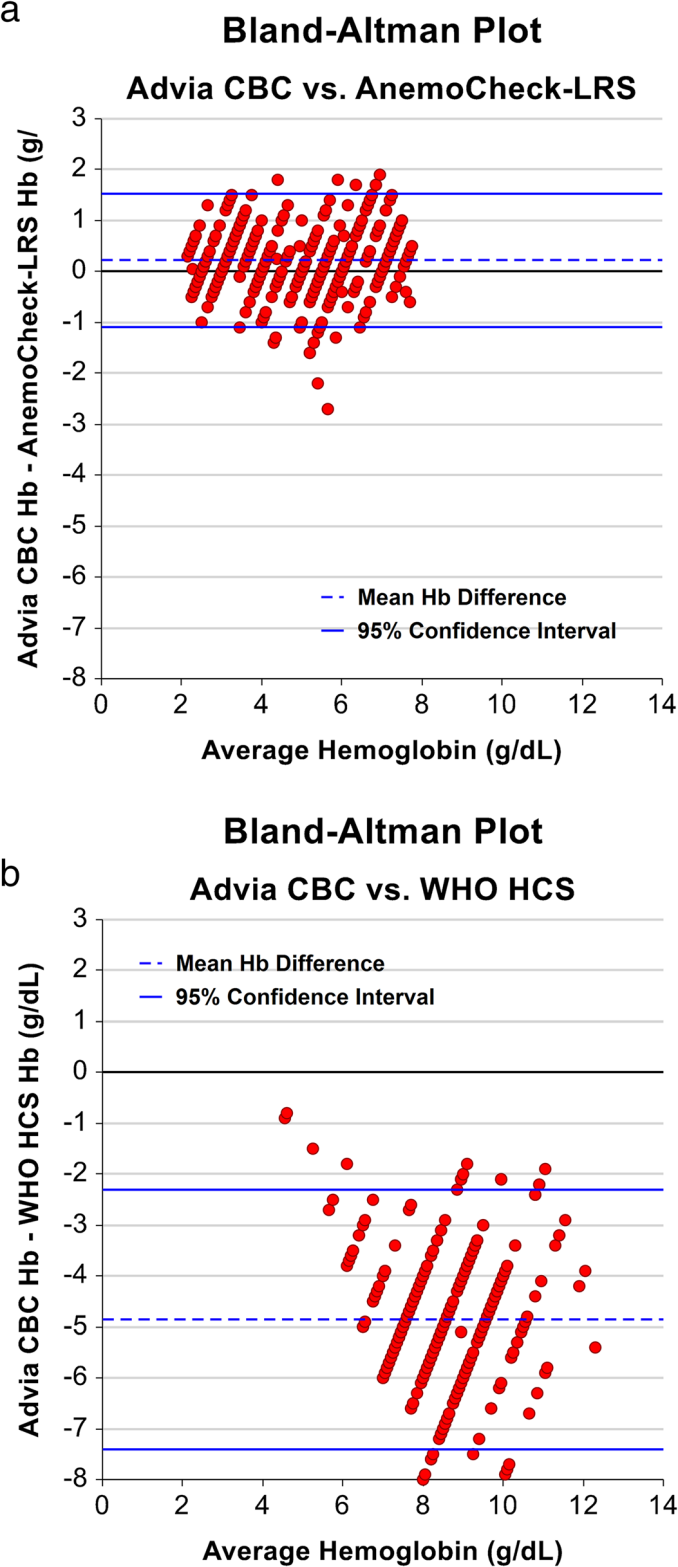
**Bland-Altman plots.** The correlation between the AnemoCheck-LRS and the gold standard method was consistent across the spectrum of measured hemoglobin values (panel **a**). Most AnemoCheck-LRS results were within 0.5 (64%) or 1.0 (86%) g/dL of the measured Hb. In contrast (panel **b**), for the WHO Haemoglobin Color Scale (WHO HCS), the correlation appeared to worsen with increasing hemoglobin values. The WHO HCS consistently overestimated Hb values by as much as 8 g/dL

A total of 600 hemoglobin measurements were recorded using AnemoCheck-LRS. This direct comparison to the measured Hb concentration included 570 measurements, excluding 30 results from the 10 samples with hemoglobin concentrations greater than 8 g/dL, which is the upper limit of detection for the AnemoCheck-LRS color scale. Of the 570 evaluations with Hb ≤ 8 g/dL, the absolute difference between AnemoCheck-LRS and measured Hb was 0.5 ± 0.4 g/dL with very strong positive correlation (*r* = 0.93, *p* < 0.0001, Fig. 3a and Table 1). Most AnemoCheck-LRS results were within 0.5 (64%) or 1.0 (86%) g/dL of the measured Hb and did not differ according to the absolute value (Fig. 4a). There was excellent consistency of interpretations by each of the three readers with an intraclass correlation coefficient of 0.96. Each of the 20 readers demonstrated a very strong correlation between their AnemoCheck-LRS values and the measured hemoglobin value, with Pearson correlation coefficient ranging from 0.86 to 0.99.

**Table 1:** Correlation matrix

	ADVIA 2120i	Hemocue Hb 201+	Anemocheck-LRS*	WHO HCS**
ADVIA 2120i	1.0			
Hemocue Hb 201+	0.99	1.0		
Anemocheck-LRS*	0.93	0.94	1.0	
WHO HCS**	0.57	0.56	0.53	1.0

*Correlation coefficients were calculated only for the limits of detection of Anemocheck-LRS (Hb ≤ 8 g/dL)

**Correlation coefficients were calculated only for the limits of detection of WHO HCS (Hb ≤ 4–14 g/dL)

### Accuracy of WHO Haemoglobin Colour Scale assay

Of the 405 measurements with Hb between 4 and 14 g/dL (the WHO HCS detection range), the absolute difference between the WHO HCS and the measured hemoglobin concentration was 4.9 ± 1.3 g/dL with moderate positive correlation (*r* = 0.57, *p* < 0.0001, Fig. 3b and Table 1). There were no HCS assessments within 0.5 g/dL and only 7 (1.7%) within 2 g/dL of the measured hemoglobin value. Overestimation of the hemoglobin concentration was noted consistently across all measured hemoglobin values (Fig. 4b). There was less consistent interpretation by each of the three readers with an intraclass correlation coefficient of 0.70 and a Pearson correlation coefficient ranging from 0.62 to 0.91.

### POC tests as a screening tool for severe and profound anemia

Recognizing that a hemoglobin concentration of 5.0 g/dL is a common threshold to consider blood transfusion, we evaluated 99 samples with a measured (ADVIA) Hb ≤ 5 g/dL and found AnemoCheck-LRS had sensitivity of 91.9% (95% CI 88.2–94.5%) and specificity of 86.1% (81.8–89.6%). For the diagnosis of WHO-defined severe anemia (Hb ≤ 7 g/dL), AnemoCheck-LRS had a sensitivity of 99% (95% CI 98–100%). Comparatively, the WHO HCS was unable to accurately identify most samples with profound anemia (Hb ≤ 5 g/dL) with a sensitivity of 27.9% (95% CI 23.2–33.3%).

### HemoCue Hb 201^+^

HemoCue Hb 201^+^ was also tested as a reference measurement to determine its utility as a reliable, portable, and accurate POC method for Hb determination. The mean absolute difference between the HemoCue and the measured ADVIA 2120i was 0.1 ± 0.2 g/dL (range 0–1.2 g/dL). Only 2% (4/200) measurements by the HemoCue were > 0.5 g/dL from the ADVIA Hematology Analyzer, suggesting that HemoCue can provide a reasonable Hb reference value in future field testing (Fig. 3c and Table 1).

### Assessment of color blindness on AnemoCheck-LRS performance

Among our readers, one individual was red-green colorblind. Colorblindness appeared to have minimal effect on his accurate reading of AnemoCheck-LRS samples (absolute difference between AnemoCheck-LRS and ADVIA CBC Hb = 0.7 ± 0.5 g/dL); there was a subjective difficulty reported when trying to assess a sample with the WHO HCS (absolute difference between WHO HCS and ADVIA CBC Hb = 6.5 ± 1.7 g/dL). For this reader, the correlation coefficient for the AnemoCheck-LRS was 0.91 (mean for all readers 0.94), whereas it was 0.61 for the WHO HCS (mean for all readers 0.82).

## Discussion

In this study, we rigorously evaluated the newly optimized AnemoCheck-LRS and focused on its ability to accurately diagnose severe (Hb ≤ 7 g/dL) and perhaps more importantly profound (Hb ≤ 5 g/dL) anemia requiring transfusion. AnemoCheck-LRS provided results that demonstrated a very strong correlation with gold standard hemoglobin measurements (*r* = 0.93, *p* < 0.0001) and was highly sensitive for detection of profound anemia (92%). Profound anemia is highly prevalent across the limited-resource settings of sub-Saharan Africa, with data from the Fluid Expansion As Supportive Therapy (FEAST) trial demonstrating that 33% of children presenting for acute care to hospitals across Africa had Hb below this 5 g/dL threshold [18, 19].

Blood is a limited commodity in most limited-resource settings and appropriate and evidence-based rationing is warranted, by appropriately identifying patients with the most profound anemia [20–23]. The timely and accurate diagnosis of anemia is an important and necessary step in determining which patients need a blood transfusion most urgently. The recently completed Transfusion and Treatment of Severe Anemia in African Children Trial (TRACT) documented a high rate of severe anemia among children in sub-Saharan Africa and the life-saving benefits of transfusions in this setting [24].

In addition to the importance of transfusing those who need it most urgently, however, clinicians must remember that blood transfusions are not without risks and should be used judiciously for those who need it. To date, the WHO HCS is the only widely available and inexpensive method of diagnosing anemia, but it has demonstrated significant variability as a true diagnostic test, particularly at very low hemoglobin levels where transfusion may be indicated [25]. WHO also recommends 2 h of training on the test before using the HCS, which may limit the ability to use this test in a wide variety of clinical settings [26]. The Anemocheck-LRS is designed to be simple and able to be used by anyone following reading the simple instructions.

Our data suggest that AnemoCheck-LRS may be a suitable replacement screening test in limited-resource settings. AnemoCheck-LRS meets the criteria for a POC test for limited-resource settings in that it is simple, rapid, and inexpensive. It is important to compare and contrast the cost and performance of Anemocheck-LRS with the HemoCue devices, which are handheld and very accurate. HemoCue devices have an upfront cost of $500 per test and require disposable cuvettes for each test that cost approximately $0.75–$2 each [27]. The test requires batteries and electricity to charge these batteries. If the handheld device malfunctions or is out of batteries, the test is unable to be performed. In contrast, AnemoCheck-LRS does not require any upfront investment, batteries, or electricity. The test is interpreted with the naked eye and does not require any electronics. If a single test fails, it can be repeated at a low cost. If Anemocheck-LRS is produced at scale (at minimum shipment volumes of 10,000), the cost could be as low as $0.30 per test. If production was increased further, this price-point could likely be reduced further. The accuracy of Anemocheck-LRS as demonstrated by these studies and the low per test cost without requirement for electricity or additional equipment are important factors that can be further validated through implementation studies to evaluate the clinical utility of this novel POC assay as a screening tool in limited-resource settings.

Our study does have important limitations. The hemoglobin range of the test (2–8 g/dL) was selected to identify severe and profound anemia that may require transfusion, but is unable to clearly identify patients with mild anemia in the Hb 9–10 g/dL range. The initial version of Anemocheck was designed for use in higher resource settings to identify mild to moderate anemia and can capture a hemoglobin range of 7.2–16.3 g/dL [15]. It is possible that two versions of this assay (severe anemia and mild/moderate anemia) may be available. This is possible by adjusting the amount of blood used in the test, but has not yet been a focus of assay development. The Anemocheck-LRS test as described, thus, does require an initial clinical assessment that raises suspicion for severe or profound anemia and is unable to detect mild anemia. The color-based nature of the scale also raises concern that those who are red-green color blind may have difficulties in interpretation. We did have a single reader who was color-blind who performed well but further assessment of this assay in color-blind persons will be important. We also recognize that the study was performed in a controlled laboratory environment in the USA. We attempted to simulate “real-life” scenarios by including many test readers with no familiarity with the test and limited laboratory experience in general. Additionally, the test used leftover venous blood and not capillary blood collected by finger stick at the point of care. Our previous studies have demonstrated that the AnemoCheck-LRS has similar results for both venous and capillary blood, but it will be important to confirm this with future field studies. In designing the current study, we recognized these limitations and intended for these data to provide important “controlled” results of the accuracy of the assay before introducing the innumerable confounding variables that may be present in field studies within limited-resource settings. Thus, if the test demonstrates less accuracy in the field, this is likely due to field-related variables and not the test itself.

## Conclusions

This study has two important conclusions. First, AnemoCheck-LRS performed very well in measuring Hb concentrations commonly found in limited-resource settings (Hb 2–8 g/dL) and, more importantly, was highly sensitive in recognizing severe and profound anemia. Although AnemoCheck-LRS performed well on its own, we analyzed the data further to identify an AnemoCheck-LRS result that could serve as a cutoff value where the likelihood of profound anemia requiring transfusion is high. We identified a slight shift in the hemoglobin color result (to the color corresponding to 6.0 g/dL in Fig. 1a) on the AnemoCheck-LRS as a “cutoff” threshold that would correctly identify 99% of cases of profound anemia where blood transfusion may be needed. The interpretation of the AnemoCheck-LRS could be reconsidered to provide a binary result rather than an exact hemoglobin estimation. For example, if the AnemoCheck-LRS color is at or below this orange color (Fig. 1a), the interpretation would be “high likelihood of requiring blood transfusion; consider transfusion if clinically indicated, or further confirmatory testing if available,” and if the color is above orange range, the result could read “low likelihood of profound anemia requiring transfusion, unless additional clinical concerns are present.” This strategy would help to rapidly identify those that may need transfusion, limit unnecessary transfusions, and reduce the number of complete blood counts performed, and thus conserve limited and expensive reagents required for these more elaborate laboratory equipment.

The second important conclusion was the superiority of AnemoCheck-LRS over the WHO HCS as a diagnostic tool for severe and profound anemia. Our data demonstrate that for samples with very low Hb levels, the WHO HCS consistently overestimated the true hemoglobin level and was an insensitive screening test for either severe (Hb ≤ 7 g/dL) or profound (Hb ≤ 5 g/dL) anemia.

In summary, this study demonstrated the possible utility of the AnemoCheck-LRS as a screening tool for severe and profound anemia in limited-resource settings. In addition, the results from HemoCue 201^+^ were very highly correlated to the gold standard and may be used as a reasonable substitute gold standard for future field studies of the AnemoCheck-LRS. These results were obtained in a controlled laboratory environment in the USA, and further field testing in limited-resource settings is necessary, including patients with various causes of anemia to ensure that the performance of the test remains strong regardless of the cause of anemia. Ideally, future studies should focus on the clinical utility of the AnemoCheck-LRS as a hemoglobin threshold screening tool to identify and manage patients with profound anemia requiring transfusion.

## Data Availability

The datasets used and/or analyzed during the current study are available from the corresponding author (patrick.mcgann@cchmc.org) on reasonable request.
